# Corrigendum to “CAMPO Precision128 Max ENERGY Spectrum CT Combined with Multiple Parameters to Evaluate the Benign and Malignant Pleural Effusion”

**DOI:** 10.1155/2021/9791042

**Published:** 2021-12-23

**Authors:** Tianyu Zhang, Cuicui Wu, Zhongtao Li, Yan Ding, Lijuan Wen, Li Wang

**Affiliations:** ^1^CT Section of the Second Hospital Affiliated Qiqihar Medical University, Qiqihar 161000, Heilongjiang, China; ^2^Qiqihar Medical University, Qiqihar 161000, Heilongjiang, China; ^3^Ultrasound Department, The Third Hospital Affiliated Qiqihar Medical University, Qiqihar 161000, Heilongjiang, China; ^4^Radiology Center, The Third Hospital Affiliated Qiqihar Medical University, Qiqihar 161000, Heilongjiang, China

In the article titled “CAMPO Precision128 Max ENERGY Spectrum CT Combined with Multiple Parameters to Evaluate the Benign and Malignant Pleural Effusion” [1], the Acknowledgments section was omitted in error. The Acknowledgments section is given as follows.
